# The role of geophagy and artisanal gold mining as risk factors for elevated blood lead levels in pregnant women in northwestern Tanzania

**DOI:** 10.1371/journal.pgph.0002958

**Published:** 2024-02-23

**Authors:** Deborah S.K. Thomas, Moses Asori, Elias C. Nyanza

**Affiliations:** 1 Department of Geography and Earth Sciences, University of North Carolina at Charlotte, Charlotte, North Carolina, United States of America; 2 Department of Environmental, Occupational and Research GIS, School of Public Health, Catholic University of Health and Allied Sciences, Mwanza, Tanzania; University at Buffalo, UNITED STATES

## Abstract

Neither artisanal and small-scale gold mining (ASGM) or geophagy practices have received substantial attention related to blood lead levels despite the well documented deleterious effects of lead. This cross-sectional analytical study aimed to document the risk of lead exposure from geophagy and mining-related occupational activities for pregnant women. The study recruited 1056 pregnant women (883 in an ASGM area and 173 in a non-ASGM area) between April 2015 –April 2017. Generalized Linear Model with an identity link function was used to model the association between blood lead levels (BLLs) and geophagy practices and involvement in gold mining. The prevalence of geophagy was 36.2% (95% CI: 33.6, 39.4%) and 6.3% engaged in mining as a primary occupation. Practicing geophagy increased BLLs by 22% (β = 1.22, 95% CI: 1.116, 1.309, p<0.0001). Living in a gold mining area increased BLLs by 33.4% (β = 1.334, 95% CI: 1.2, 1.483, p<0.0001). Having mining as a primary occupation increased BLLs by 1.3% β = 1.013, 95% CI: 0.872, 1.176, p = 0.869) even though the association was not statistically significant. Socioeconomic wealth quantile (β = 1.037, 95% CI: 1.021, 1.054, p<0.001) increased blood lead levels by 3.7%. Developing a comprehensive inventory capturing sources of community-level lead exposure is essential. Further, increasing public health campaigns and education are crucial to limit geophagy practices and to minimize work in gold mining activities during pregnancy.

## 1. Introduction

Despite widely documented health risks from lead (Pb) exposure that span neurological impairments, immunological, cardiometabolic to reproductive health risks, it persists as a significant and overlooked global environmental health challenge. Annually, the WHO estimates that one million people perish from lead exposure [1], but the effects are more far reaching. Even though there is no safe Pb level according to the US CDC and WHO [1, 2], averages in Sub-Saharan Africa (SSA) commonly exceed the World Health Organization’s (WHO) recommended concentrations of 10 μg/L for drinking water [3] and 0.5 μg/m3 for Pb in air [4]. For example, a continental study investigating of Pb levels in drinking water in Ghana, Mali, and Niger found that 9% exceeded the WHO recommended levels [5].

Due to its ability to displace calcium in the human body, Pb presents significant neurotoxicity in the central nervous system (CNS) and peripheral nervous system (PCNS), increasing the risk of encephalopathy and cognitive impairment [6]. Other notable health outcomes from Pb exposure include hemolytic anemia, hypertension, and cardiovascular disease [7–10]. Prolonged and cumulative exposures from childhood have also been associated with reduced libido, abnormal spermatogenesis, decreased fertility in men, and miscarriage, premature delivery, cognitive and intelligence declines [11–13]. Since pregnant women may transmit Pb to their unborn children in-utero due to its ability to cross the placenta [6], the health effects may be inter-generational with varied health implications in later years [7, 11–13]. One study that compared maternal blood, cord blood, and the amniotic fluid Pb levels among nineteen women at delivery found higher concentration in the amniotic fluid than in the cord blood [14]. The data also showed higher levels in the fetal membrane, suggesting Pb may have been absorbed from the amniotic fluid. Recent epidemiological evidence also suggests that Pb exposure may be associated with aggression and social deviancy among children and youths [15]. Acute lead poisoning may even result in instant death. Unborn babies who are maternally exposed to lead are more likely to develop neurological disorders and impaired cognitive development due to their liver’s inability to detoxify such toxic heavy metals [16].

A systematic review of blood Pb levels among women of reproductive age in Africa showed that the weighted mean of blood levels ranged from 0.83 to 99 μg/dl, with an overall mean of 24.73 μg/dl, whereas the average among pregnant women was 26.24 μg/dl [17]. The averages reported were 4.8–5 times higher than the threshold limit of 5μg/dl set by the WHO and CDC. This elevated exposure may be explained by the diverse exposure pathways including artisanal gold mining activities, smelting, lead-battery processing, e-waste processing, urban vehicular and industrial emissions, and exposure to Pb paint. These diverse sources of exposure coupled with low awareness of lead exposure hazards, high rate of poverty, and inadequate regulation of lead in consumer products [17], makes the impact significantly lethal and multi-dimensional. Even though data on hospitalization and loss of Gross Domestic Product (GDP) are not well-documented, a study by Attina et al in 2013 [18], estimated that $134 billion USD had been lost due to childhood Pb exposure in Africa.

The association between artisanal gold mining and geophagy practices during pregnancy and Pb risk has received minimal attention. Deliberate ingestion of soil (geophagy) among women during pregnancy or breastfeeding is widespread in Sub-Sahara Africa (SSA) [19–21]. Nevertheless, evidence from the literature suggests that geophagic materials contain environmentally persistent toxic heavy metals such as Pb [21]. While Pb is naturally occurring, human activities including mineral extraction may increase environmental pervasiveness and human exposure [22]. In areas where artisanal small-scale gold mining (ASGM) activities are pervasive, Pb contamination in soil has been reported [22]. As such, pregnant women who live proximate to mining centers and regularly practice geophagy are likely to be at greater health risk, including their unborn babies.

Ample evidence suggests geophagy practices are widespread in SSA [19–21], with prevalence ranging from 10% to 75% [23–25]. Since geophagy materials may be acquired from many sources, including soil sticks in the local market, termite mounds, and the ground [20, 26], they may be contaminated with Pb and other heavy metals. Even though some benefits of geophagy practices (supplication of micronutrients for reducing gestational nausea or detoxifying foods) have been reported [26, 27], the elevated propensity for Pb contamination makes it a health risk to both the mother and the unborn child. In addition, besides lead-based reagents and chemicals used in gold extraction, Pb may co-occur with gold and other minerals naturally [28–31]. Even with substantial evidence of the deleterious effects of Pb, limiting exposure is not an environmental health priority in SSA. For example, even though there was a legislative reform in South Africa to increase the inclusivity of women in mining sectors, little was done to evaluate the scale and intensity of occupational hazards, including environmental heavy metals exposure [32], and the story is similar in many other countries including Tanzania.

Women who are co-exposed to Pb through ASGM activities and geophagy practices may have significantly higher exposure. Given the detrimental health effects coupled with relative limited research, this study assesses geophagy and ASGM activities associated Pb co-exposure to pregnant women, building on previous work in Geita District, northwestern Tanzania. We hypothesize that women who live in close proximity to ASGM areas, work in the ASGM, and/or practice geophagy may have elevated blood Pb levels due to co-exposures as compared to women who live in non-gold mining areas, who do not work in the mines, and do not practice geophagy.

## 2. Materials and methods

### 2.1 Study design, study setting, and study population

This cross-sectional analytical study is part of the ongoing Mining and Health Prospective Longitudinal Study in Northern Tanzania. This cohort’s details and main characteristics have been explained elsewhere [33, 34]. Briefly, the study involved two districts (Geita District, an area with artisanal and small-scale gold mining (ASGM) activities, and Magu District, a non-ASGM area comparison group) with almost similar socio-economic activities except gold mining. They are distanced by 160km by road, or 150km by the radial distance with a population of 407,144 and 153,298 women respectively [33]. The recruitment, exclusion, and eligibility of pregnant women in ASGM and non-ASGM areas has been detailed elsewhere [34]. Briefly, a total of 1078 pregnant women attending antenatal care clinics in their second trimester (16–27 weeks gestation age ‐ calculated from the woman’s last menstrual period) were eligible for the study provided they had lived in the study areas for more than six months. Most of the women in northwestern Tanzania do not access antenatal care until late in their first trimester or into their second trimester [35], so that recruitment in the first trimester is challenging. At the local antenatal care clinics, public health nurses were trained in the administration of the questionnaire for interviewing the recruited pregnant women. The BLL were not known at the time of the assessment as samples had to be taken to an accredited laboratory and the results were made available to the participants after a month. In total, 1056 consented to participate (883 from Geita and 173 from Magu) between April 2015 –April 2017. Pregnant women were excluded due to medical conditions, failure to provide laboratory samples and failure to complete the face-to-face interviews (See S1 Fig).

### 2.2 Ethical considerations

Since this study involved a vulnerable population and biological materials, several research and ethics reviews and approval were obtained. The first ethics review and approval was granted by the Joint Catholic University of Health and Allied Sciences and Bugando Medical Centre’s Ethics and Research Review Committee (*BREC/001/38/2014-2024*). The protocol was also reviewed and approved by the Tanzania National Institute for Medical Research (*MR/001/38/2014*). Permission to conduct the study was obtained from the relevant health authorities at regional and district levels in ASGM and non-ASGM areas. Pregnant women attending antenatal care clinics were given study information, including a brief introduction regarding the study team and scope, benefits, and potential risks of participating before recruitment. A written informed consent was obtained among individuals who participated in this study. The consent form was written in Kiswahili, the primary language of most of the population of Tanzania, which explained the purpose, potential risks, significance, and the right to participate or withdraw from the study. Once they decided to participate, they signed a thumbprint indicating their consent form. The consent form was also read for individual women with low literacy. Individual pregnant women who were younger than 18 years of age provided a written assent, and their parents/guardians provided informed consent. During the consenting process, an impartial witness from the local community was present to ensure complete autonomy in the decision-making process. Their participation did not impact the care and services participants received at the health facility as detailed elsewhere [33]. Special codes were used instead of names to protect participants’ information. Once laboratory results on blood lead levels were available, efforts were made to inform women about their exposure status.

### 2.3 Maternal blood lead concentrations

A dried blood spot sample (DBS) was collected among pregnant women in this study during 16 to 27 weeks of gestation [33, 36]. The DBS method validation for accuracy, validity, and reliability has been detailed elsewhere [35]. Briefly, DBS were collected on filter paper (Whatman903) following a simple finger prick [33–36], air dried in a closed desiccator overnight, and packed into trace metal–free Nalgene resealable plastic bags for more extended storage in a desiccator until they were shipped to the laboratory [33, 36].

Several quality control measures were included. *1*^*st*^ ‐ running laboratory blanks, n = 26, field blanks, n = 26 samples (i.e., blank Whatman #903 filter papers) were included to account for potential environmental contamination during sampling and analytical procedures. *2*^*nd*^ ‐ a sample of DBS (n = 44) was spared to venous blood samples from the same participant, where a strong correlation (r^2^ > 0.9) and a slope close to 1.0 between DBS concentration and quantitative venous blood indicated high accuracy of the method [36]. *3*^*rd*^ ‐ laboratory Seronorm whole blood reference materials (SRM, Seronorm trace elements whole blood L‐2, lot 1406264; Sero) were digested and run before and after every batch of 10 samples [36]. *4*^*th*^ ‐ in every batch of 20 DBS samples, a replicate second sample from one of the participants was analyzed under identical laboratory conditions (preparation/digestion/analyses). *5*^*th*^ ‐ to avoid categorizing unexposed pregnant women to the exposed group due to measurement errors (i.e., exposure misclassification), method detection limit (MDL) (0.08 μg/L for Pb) was determined based on field blanks (i.e., mean + 3 times standard deviation) [33]. DBS samples underwent microwave-assisted closed vessel acid digestion at 170°C for 30 min [33, 36]. Blood total Pb (T-Pb) concentrations were determined using inductively coupled plasma mass spectrometry (ICP-MS; PerkinElmer, Shelton, CT, USA) (μg/L) [35, 37, 38], at an ISO 17025 accredited laboratory (ALS *Scandinavia*, Sweden) [33, 35]. Multiple isotopes were also monitored, and all results were within a 10% relative standard deviation, indicating the precision of the chemical analysis [35].

### 2.4 Explanatory variables

Pregnant women completed a structured self-report questionnaire administered via face-to-face interviews during antenatal care visits that captured maternal age, education, maternal and paternal occupation (including working in a mine), and family socioeconomic status (*See S1 Data*). Socio-economic status was established similar to the Demographic and Health Survey [35], which measures asset ownership (e.g., ownership of house or bicycle, home sanitation facilities) to estimate participants’ family socioeconomic wealth quintiles (SEWQ) [39]. Briefly, eight assets to classify women’s SEWQ based on the Tanzanian context were used as detailed in [33, 35, 38], including ownership of a house, materials used for housing construction, access to water for domestic use, access to electricity or solar energy at the household, number of meals usually eaten per day, permanent source of income, partner’s sources of income, and ownership of assets for transport such as bicycles, motorcycle or car [33, 35]. Total scores on the SEWQ were categorized as high (>9 scores), moderate (6–9), or low (<6) SES [33]. Geophagy was captured by asking if they ate or had a history of eating soil during pregnancy. All associations were considered statistically significant at a 5% significance level. Previously, we reported that maternal age, maternal education, marital status, maternal occupation, and family socioeconomic status (SES) were associated with maternal exposure to As and Hg in this cohort [33].

### 2.5 Statistical analysis

Non-transformed median BLLs between sub-groups were compared using the Mann-Whitney U test. A generalized linear model with an identity link function was used to estimate the association between blood lead levels (BLLs) with occupational exposure and geophagy practices. Due to skewed data distribution, the Kruskal-Wallis test was used to analyze the median differences between groups for lead concentration. The dependent variable was the logged BLLs for pregnant women and the independent variables included geophagy practices, involvement in artisanal gold mining, area of residence, and other socio-demographic characteristics. Due to skewed distribution, BLLs were log transformed to adjust for normality and homogeneity assumption for regression analysis. For appropriate interpretation, log transformed BLLs results from univariate and multivariable linear regression were exponentiated. The impact of geophagy practices on BLLs were adjusted for age, education level, mining residence, occupation, and socioeconomic status. A p-value of < .05 was considered statistically significant.

## 3. Results

The average age of the 1056 pregnant women was 25.5 (SD = 6.3) years, and most (n = 397; 32.1%) were between 20–24 years of age. A majority (71.6%) had as least a primary school education, while only 8.3% had a high school education or higher. Mining comprised 6.3% of their primary occupation, with 5.8% in business and a majority (n = 905; 85.7%) in agriculture. Most women (78.3%) lived in the study areas for more than five years. Most women (50.4%) were in the medium socioeconomic wealth quantile range, with 18.9% in the high category and 30.7% in the low SEWQ (Table 1).

**Table 1:** Socio-demographic status among the study participants (N = 1056).

Variables	n = 1056	(SD /%)
BMI^€^ of the mother (mean, SD) kg/m^2^	24.4	(3.4) ^*¥*^
Average age of pregnant women (mean, SD) years	25.5	(6.3)
The age group of mothers		
14–18	128	(17.6)
20–24	397	(32.1)
25–29	255	(24.2)
30–34	157	(14.9)
35–39	91	(8.6)
40+	28	(2.6)
Mother education		
None	210	(19.9)
Primary education (7 years)	756	(71.6)
Secondary and above (> 7 years)	90	(8.3)
Partner education		
None	132	(12.5)
Primary education (7 years)	797	(75.5)
Secondary and above (> 7 years)	127	(11.6)
Mother has had vocational training		
Yes	129	(12.2)
No	927	(87.8)
Years of residence in a respective district		
< 2	28	(2.7)
2–5	201	(19.0)
5+	827	(78.3)
Marital status		
Married	868	(82.2)
Cohabiting	135	(12.8)
Single/Separated/Divorced	53	(5.0)
Mother’s occupation		
Farm/animal keeping	905	(85.7)
Mining	72	(6.3)
Business	61	(5.8)
Public servant	18	(1.7)
Partner’s occupation		
Farm/animal keeping	683	(64.7)
Mining	285	(26.1)
Business	61	(5.8)
Public servant	27	(2.5)
Social Economic Wealth Quintile (SEWQ^¥¥^)		
High	200	(18.9)
Moderate	532	(50.4)
Low	324	(30.7)
Where soil is obtained		
Ground soil	177	(46.1)
From the market	207	(53.9)

Note: ^¥^Standard Deviation (SD)

^€^Body Mass Index (BMI) determined using body weight in kilograms and square of their respective height in meters; and

^¥¥^Social Economic Wealth Quintile (SEWQ) ‐ assessed based on ownership of different assets among women’s households).

**Fig 1:**
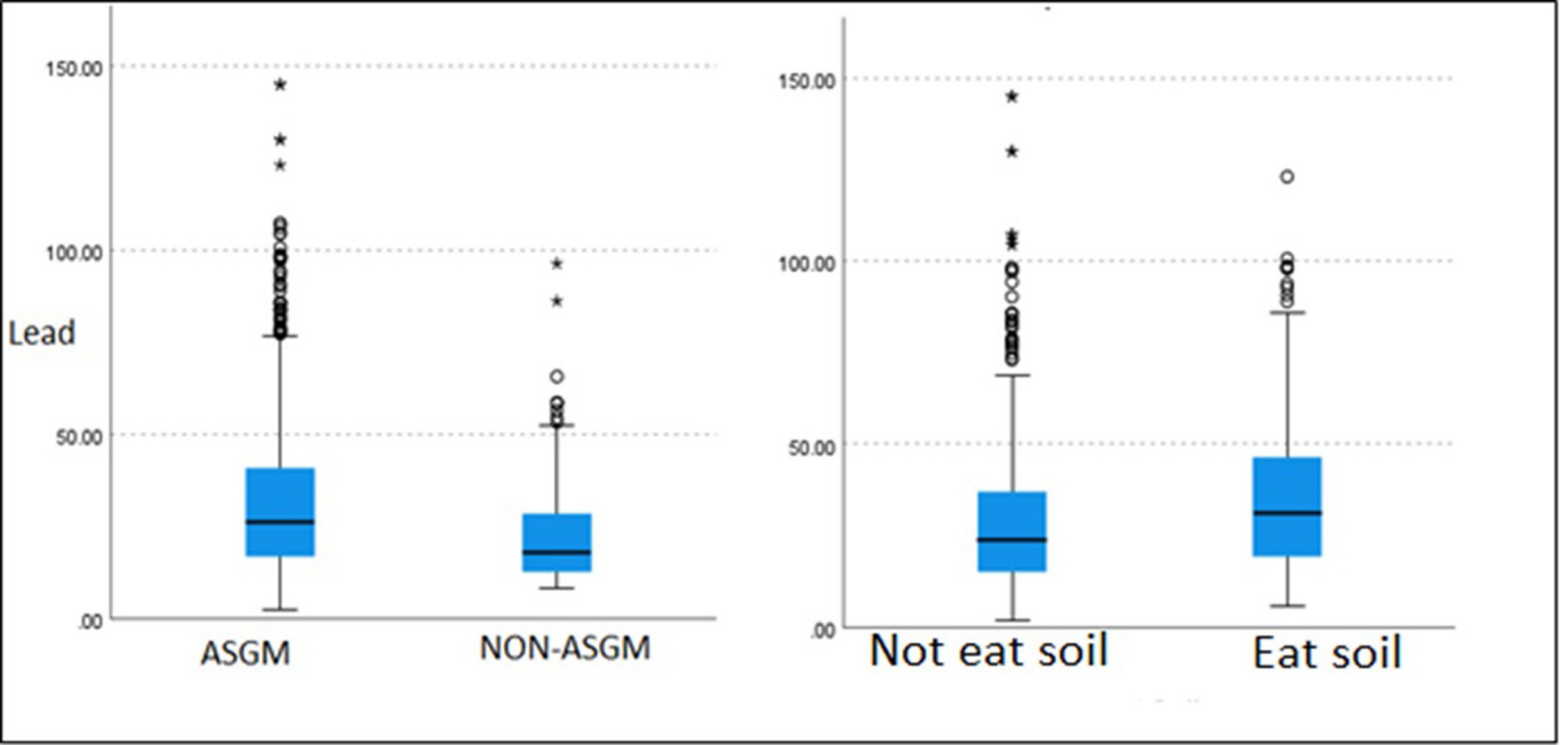
A box plot showing the distribution of lead levels based on location and geophagy practices.

The prevalence of geophagy was 36.2% (95% CI: 33.6, 39.4%). However, 53.91% (95% CI: 48.7, 59.1%) reported obtaining their soil from the market, whereas 46.1% (95% CI: 40.9, 51.3%) obtained theirs from the ground. Table 2 and Fig 1, provides a detailed description of BLLs based on geophagy practice and whether they lived in a mining community. Overall, there were statistically significant differences in median Pb concentration between women who practiced geophagy and those who did not work in mining areas (χ^2^ = 26.833, p<0.0001). Women in mining areas had significantly elevated BLLs than those in non-mining areas regardless of their geophagy practices and occupation (χ^2^ = 46.076, p<0.0001). Among those who did not practice geophagy, working in mines was associated with increased BLLs (χ^2^ = 32.515, p<0.0001).

**Table 2:** Descriptive details of lead distributions based on occupation, geophagy practices, and location differences (N = 1056).

**Lead Concentration (**μg/L**)**			
	Median	IQR/%	
**AGSM Community**			
Practicing geophagy	31.06	19.37–46.47	
Not practicing geophagy	23.94	15.14–37.03	
Working in mining (overall)	27.22	17.93–42.81	
Not working in mining (overall)	20.32	13.02–34.47	
Working in mining (geophagy)	31.01	19.33–46.39	
Working in mining (no geophagy)	25.74	15.66–40.01	
Not working in mining (no geophagy)	15.13	11.88–23.01	
**Group comparison**			
Comparison	Median difference	Test-statistic	p-value
Geophagy practices vs. non-geophagy	7.12	χ^2^ = 26.83	<0.0001
Geophagy in non-Mining areas vs. non-geophagy in mining areas	20.19	χ^2^ = 38.45	<0.0001
Mining vs. non-mining area (overall)	8.26	χ^2^ = 46.08	<0.0001
Working in mine vs. not working in mine with geophagy practices	3.38	χ^2^ = 0.05	0.942
Working in mine vs. not working in mine without geophagy practices	10.61	χ^2^ = 32.51	<0.001
**Non-AGSM Community**			
Practicing geophagy	29.59	16.62–44.68	
Not practicing geophagy	16.66	11.77–21.82	
**Group comparison**			
Geophagy practices vs. non-geophagy	12.93	χ^2^ = 13.14	<0.001
Not eating in non-Mining areas Vs. Not eating in mining areas	7.26	χ^2^ = 92.69	<0.001
Mining vs. non-mining area median difference	8.26	χ^2^ = 46.08	<0.001

**Note:** Artisanal and small-scale gold mining (ASGM) includes formal or formal, mostly undercapitalized, and under-equipped gold extraction operations where technical and management skills are lacking and environmental management is inadequate or minimal.

Among those women who practiced geophagy, working in mining was not associated with a median difference in BLLs (χ^2^ = 0.0047, p = 0.945193). There was a statistically significant median difference in Pb levels between those who did not practice geophagy in mining areas and those who did in non-mining areas (χ^2^ = 38.452, p<0.0001). In non-gold mining areas, those who practiced geophagy still had elevated levels of Pb as compared to those who did not practice (χ2 = 13.137, p<0.001). Among pregnant women who did not practice geophagy, living in mining areas was linked with higher levels of Pb (χ^2^ = 13.137, p<0.001).

Adjusted and unadjusted models of the association between geophagy practices and lead concentration are detailed in Table 3. In the unadjusted model, pregnant women who practiced geophagy had increased Pb levels 33% more than those who did not practice (β = 1.333, 95% CI: 1.236, 1.437, p<0.0001). Living in mining areas was associated with an approximately 39.4% increase in Pb levels (β = 1.394, 95% CI: 1.276, 1.524, p<0.0001). There was no statistically significant association between educational attainment and BLLs (β = 0.939, 95% CI: 0.84, 1.038, p = 0.217). Working in mines (β = 1, 95% CI: 0.862, 1.161, p = 0.998) and where the soil was obtained (β = 1.048, 95% CI: 0.932, 1.178, p = 0.433) were also not associated with BLLs in pregnant women. However, age increased the concentration by 6% (β = 1.006, 95% CI: 1.00, 1.013, p = 0.036). Socioeconomic wealth quantile was positively associated with BLLs (β = 1.031, 95% CI: 1.016, 1.047, p < .001).

**Table 3:** Association between geophagy practices among pregnant women and blood lead concentration (N = 1056).

Lead concentration (μg/L)						
Explanatory Variables	Unadjusted			Adjusted		
	exp(coefficient^β^)	(95% CI)	p-value	exp (coefficient^β^)	(95% CI)	p-value
The age group of mothers (years)	1.006	(1.00, 1.013)	0.036*	1.003	(0.995, 1.012)	0.455
*Mother education*						
At least primary education	1			1		
No formal education	0.939	(0.84, 1.038)	0.217	1.278	(1.118,1.461)	<0.0001
*History of eating soil during pregnancy*						
No	1			1		
Yes	1.333	(1.236, 1.437)	<0.0001***	1.229	(1.094,1.380)	<0.0001
*Living in the ASGM area*						
No	1			1		
Yes	1.394	(1.276, 1.524)	<0.0001***	1.212	(1.039,1.414)	0.014
*Working in mining*						
No	1			1		
Yes	1.000	(0.862, 1.161)	0.998	1.090	(0.877, 1.355)	0.431
*Where soil is obtained*						
Ground soil	1.048	(0.932, 1.178)	0.433	1.056	(0.862, 1.674)	0.532
From the market	1			1		

**Note:** ASGM — Artisanal and small-scale gold mining. For the unadjusted model: association between BLL and each predictor independently. For an adjusted model: adjusted by maternal age, education, living in the mining area, working in mines, and where the soil is obtained.

In the adjusted model, practicing geophagy increased BLLs by 22% (β = 1.22, 95% CI: 1.116, 1.309, p<0.0001). Living in a gold mining area increased BLLs by 33.4% (β = 1.334, 95% CI: 1.2, 1.483, p<0.0001). Age of respondents (β = 1.005, 95% CI: 0.999, 1.011, p = 0.097) and socioeconomic wealth quantile (β = 1.037, 95% CI: 1.021, 1.054, p<0.001) increased BLLs by 0.5% and 3.7% respectively. However, educational attainment (β = 0.966, 95% CI: 0.881, 1.059, p = 0.457) and working in the mines as a primary occupation (β = 1.013, 95% CI: 0.872, 1.176, p = 0.869) did not predict BLLs significantly.

## 4. Discussion

The prevalence of geophagy practices among pregnant women in this study (36%) is consistent with an SSA prevalence of 36–56%. In our study, half of the women who ate soils obtained them from the ground. Ground soil has been noted to be severely contaminated with multiple heavy metals [20, 26, 40]. Despite consistent evidence of higher toxic chemical element exploration from practicing geophagy, the motivations and drivers of the practice are complex [41],

Increased maternal Pb risk from geophagy practices and ASGM mining operations in our study demonstrates a critical need for surveillance and risk reduction from ecological and occupational Pb exposures. Pregnant women living in mining areas and who practiced geophagy had the highest Pb levels. Given that there is no safe Pb level [27, 42], the levels found in this study were alarming. Those who ate soil during pregnancy had increased Pb levels by 33% and those living in mining communities had a 34% increase in Pb levels. Our findings among pregnant women who practice geophagy are higher than those reported in Johannesburg, South Africa (median 31.1 vs 21.0 μg/L, respectively) [43], but are lower compared to those reported among mothers in Benin (>50.0 μg/L) where the sources of lead were reported to be drinking water and meat from animals killed by ammunition [44]. These findings are extremely concerning given the potentially harmful effects of these exposures on unborn babies, such as cognitive impairment and neurological disorders [20, 33, 45].

The realization that the above- Pb levels exist in most SSA countries is not necessarily new [46]; and in-utero toxicity was reported over a decade ago [47]. However, the ways in which geophagy practices, living in mining communities, and working in mines coalesce to increase Pb risk for pregnant women is poorly documented in SSA. Studies have indicated that Pb bioaccumulates in calcium-rich tissues, including bones [27, 47, 48]. In-utero exposure may lead to irreparable neurological disorders and cognitive malfunctioning throughout the life-course of children whose mothers were significantly exposed [27]. Further, calcium and iron deficiency and malnutrition have been associated with elevated levels of Pb [27]. Future studies should include nutritional conditions, calcium, and iron deficiencies in association to levels of Pb among the study participants.

Crucially, because living in mining areas was significantly associated with increased Pb levels for the women in this study, the potential of Pb risk for all people in these mining communities is highly likely. ASGM contaminates the environment with Pb through transport in water bodies and air, as has been documented in Ghana and other countries [49, 50]. In South Africa, pregnant women residing near mining areas were reported to have elevated BLL as compared to those who reside in non-mining rural areas (mean BLL 26.4 vs 20.9 μg/L) [51]. Of significant concern are children who play and eat soil from the ground. Combined effects of occupation and community exposures may elevate the cumulative health risk, particularly when combined with geophagy.

Environmental management policies that take the local context and community into consideration should be formulated and enforced to lessen ecological and occupational contamination to Pb, especially from gold mining operations. Surveillance and testing of chemical constituents of soil sticks sold in the market, including public education, would also be useful in limiting exposure to Pb, as suggested by earlier studies [26]. Increased maternal occupational Pb exposure underscores the need to improve socioeconomic conditions and increase/diversify employment options for the people in ASGM communities in Tanzania.

The strength of this study is its use of direct assessment of maternal blood lead levels in contrast to the use ecological exposure. Use of ecological data such as drinking water, soil, or plants contamination, are said to either overestimate or underestimate the actual risk of the exposure [52]. In addition, the large sample size (n = 1056), in this study provided significant power to detect and quantify the risk of BLL among the study participants. A comparison group of pregnant women from non-ASGM communities increased the robustness and confidence of the findings about the risk of BLL in mining communities of northwestern Tanzania.

This study has several limitations. Most importantly, sources of lead were not established through the testing of water, air, food, or soil, or even soil sticks sold in the markets Evidence suggests soil sticks are a source of Pb. Nyanza et al. [26], reported up to 7.4mg/kg in soil sticks and up to 11.3mg/kg in ground soil identified to be eaten by pregnant women. Concentration of lead levels is reported to differ based on the geophagia materials sources [24, 26]. The reported BLLs among pregnant women in areas without ASGM activities suggests a possibility of Pb exposure from sources other than gold mining, including soil sticks sold in the market. Community-level Pb exposure pathways for effective intervention may include soil, air, lead based paints, leaded drinking water pipes, disposed lead batteries, and many others, which need to be examined.. Developing a comprehensive inventory to target multiple sources of community-level Pb exposure is essential for reducing associated health risks.

Findings from this study cannot be generalized to other settings as sources of Pb are diverse The excluded women (n = 22) may have introduced some potential selection bias, although the large sample size (n = 1056) provides an added advantage. The use of standardized unvalidated questionnaire without a comprehensive assessment of geophagy or other potential exposure sources could have reduced the reliability of our findings. Lastly, there is a potential for recall bias which could have attributed to exposure misclassification, as geophagy is determined based on current as well as historical recall. An attempt was made to restrict recruitment during the second trimester so as to minimize the recall bias.

## 5. Conclusion

High maternal Pb levels among study participants presents significant health risks to both unborn babies and pregnant women. Importantly, the study found those who did not practice geophagy, did not work in artisanal mining, and did not reside in gold mining areas had substantially reduced BLLs. Ecological and community exposure pose a significant maternal health risk, especially in ASGM areas, regardless of whether these women engage in artisanal gold mining activities or practice geophagy. Immediate and intensive public health campaigns, education, and development and enforcement of policy for reducing lead exposure is essential, particularly for the protection of pregnant women and their unborn children.

## Supporting information

S1 Fig Recruitment, exclusion, and eligibility of pregnant women in ASGM and non-ASGM areas. (TIF)

S1 Data De-identified data and detailed information regarding the study participants. (CSV)

S1 Checklist Inclusivity-in-global-research-questionnaire. (DOCX)

S2 Checklist STROBE statement—checklist of items that should be included in reports of observational studies. (DOCX)
