# Supplementary figures and images for: The role of geophagy and artisanal gold mining as risk factors for elevated blood lead levels in pregnant women in northwestern Tanzania
Source: PLOS Glob Public Health. 2024 Feb 23;4(2):e0002958. doi: 10.1371/journal.pgph.0002958 (PMC10889643; doi:10.1371/journal.pgph.0002958)

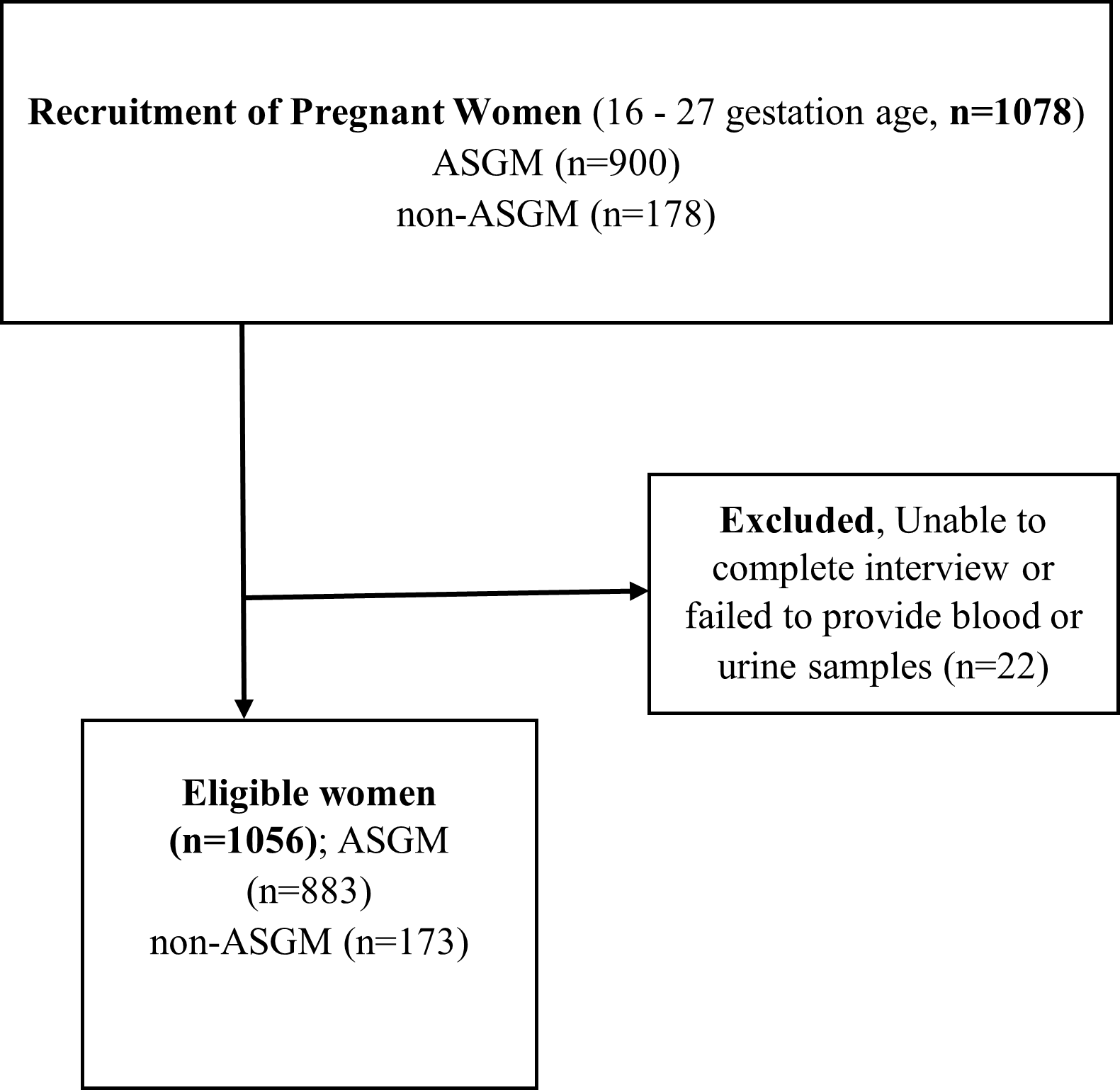

Supplement: S1 Fig — (TIF) [file pgph.0002958.s001.tif]
